# First Steps into Ruminal Microbiota Robustness

**DOI:** 10.3390/ani12182366

**Published:** 2022-09-11

**Authors:** Sandra Costa-Roura, Daniel Villalba, Joaquim Balcells, Gabriel De la Fuente

**Affiliations:** Department of Animal Science, University of Lleida, 25198 Lleida, Spain

**Keywords:** rumen, microbiota, disturbance, robustness, alpha diversity, network complexity

## Abstract

**Simple Summary:**

Microbial fermentation in the rumen is of central importance with respect to ruminant nutrition, as it provides energy and protein to meet animal requirements. The development of high-throughput sequencing techniques has provided an opportunity to appreciate that rumen microbial community is very diverse, making it difficult to define its normal status; alternatively, the concept of robustness, understood as the ability of a community to cope with disturbances, has been proposed as a marker of a healthy microbiota. The aim of the present review is to define the concept of microbial community robustness, with special focus on ruminal microbiota, and to describe its potential drivers. The robustness of the microbiota depends on its resistance, resilience and functional redundancy and is enhanced by increased alpha diversity and network complexity. Diverse feeding practices can shape the microbial community in the rumen and ultimately affect its robustness; a high-forage diet seems to be the most reliable strategy to increase both alpha diversity and network complexity and to improve robustness. Additional research should be conducted to confirm the link between microbial community robustness in the rumen and animal health and to elucidate the practical benefits of building a robust rumen.

**Abstract:**

Despite its central role in ruminant nutrition, little is known about ruminal microbiota robustness, which is understood as the ability of the microbiota to cope with disturbances. The aim of the present review is to offer a comprehensive description of microbial robustness, as well as its potential drivers, with special focus on ruminal microbiota. First, we provide a briefing on the current knowledge about ruminal microbiota. Second, we define the concept of disturbance (any discrete event that disrupts the structure of a community and changes either the resource availability or the physical environment). Third, we discuss community resistance (the ability to remain unchanged in the face of a disturbance), resilience (the ability to return to the initial structure following a disturbance) and functional redundancy (the ability to maintain or recover initial function despite compositional changes), all of which are considered to be key properties of robust microbial communities. Then, we provide an overview of the currently available methodologies to assess community robustness, as well as its drivers (microbial diversity and network complexity) and its potential modulation through diet. Finally, we propose future lines of research on ruminal microbiota robustness.

## 1. Introduction

Rumen is one of the most extensively studied gut ecosystems owing to the importance of ruminants for human nutrition. Rumen microbial fermentation is of central importance for ruminant nutrition, as it provides energy in the form of volatile fatty acids and protein in the form of microbial cell protein to meet animal requirements [1]. Owing to this specialized microbial fermentation, ruminant animals can provide humans with valuable products, such as meat, milk and wool, derived from marginal land without competing with food crop production [2].

The study of ruminal microbiota started with the development of culture techniques suitable for strictly anaerobic bacteria; however, microbiologists soon realized that cultivable isolates constituted a small proportion of the total bacterial community in rumen (around 8% [3]). The development of high-throughput sequencing techniques has provided an opportunity to fully appreciate the wide variety of microbial species inhabiting the rumen [4,5]. Despite the significant advancements in characterization of the composition and abundance of ruminal microbes, their function and interactions are yet to be fully understood. Ruminal microbiota robustness is no exception; the ability of the rumen microbial community to cope with disturbances remains largely unknown. 

The aim of the present review is to define the concept of robustness in microbial communities and to describe currently available assessment methodologies, as well as its potential drivers.

## 2. Briefing on the Current Knowledge about Ruminal Microbiota

The rumen is a complex ecosystem in which anaerobic bacteria, protozoa, anaerobic fungi, methanogenic archaea and phages act synergistically to ferment plant structural and nonstructural carbohydrates and proteins.

Bacteria are the most abundant and diverse microbial group in the rumen, and as a whole, they exhibit numerous enzymatic activities to carry out digestion of starch, plant cell walls, protein and lipids [6]. Bacterial abundance has been reported to be as high as 10^12^ individuals per gram of rumen content [7], and bacterial diversity is estimated to be around 7000 species of 19 existing phyla, with a clear predominance of Firmicutes (56%), Bacteroidetes (31%) and Proteobacteria (4%) [8]. 

Archaea are responsible for methane production in the rumen, which is then eructed and released into the environment; methane is produced mainly via the hydrogenotrophic pathway or, less commonly, through the methylotrophic and acetoclastic pathways [9,10]. Archaea abundance varies between 10^8^ and 10^10^ gene copies per gram of rumen content [11], mostly ascribed to genera *Methanobrevibacter* (>60%) and *Methanomicrobium* (15%) and rumen cluster C (16%) [8]. 

The role of protozoa has attracted considerable interest; they stabilize rumen pH when animals are fed diets rich in available starch, take part in the initial stages of fiber colonization, exerting a positive effect on fiber degradation and produce H_2_, which subsequently serves as substrate for methanogens to reduce CO_2_ to methane [12]. Protozoa abundance in the rumen is approximately 10^5^ and 10^6^ cells per gram of rumen content, with *Entodinium* as the most dominant genus [7].

Anaerobic fungi are known to participate in fiber degradation in the rumen; on the one hand, they possess an extensive set of enzymes for the degradation of plant structural polymers [13]; on the other hand, their rhizoids physically penetrate and break down the plant cell wall, increasing the surface area available for colonization by other ruminal microbes [14]. The abundance of fungi in the rumen is estimated to be around 10% of the total microbial biomass [15], and the genus *Piromyces* is usually the most represented species in the herbivore gut ecosystem, although it has been established that many other fungal taxa remain uncharacterized [16]. 

The presence of lytic phages in ruminal fluid was first observed in 1966 [17]; however, their specific effects on ruminal microbiota and fermentation remain to be determined [6]. 

The composition of the ruminal microbiota throughout life is shaped by both deterministic factors, such as diet and age, and stochastic factors, such as early colonization events. Rumen microbial communities are established soon after birth in a sequence implying a decrease in aerobic and facultative anaerobic taxa and an increase in anaerobic taxa; these early-arriving species can act as modulators of microbial community composition, hindering or facilitating the establishment of late-arriving species [18]. As a ruminant animal ages, the microbial diversity and within-age-group similarity generally increase, suggesting the establishment of a more diverse but homogeneous and specific mature community as compared with the more heterogeneous and less diverse primary community [19,20]. 

In recent years, literature has described that although the ruminal microbiota is very diverse and variable, a core microbiome exists across ruminants, depending on the diet, host and age [19,21]. The core microbiota comprises highly adapted species that usually colonize the rumen soon after birth and have the ability to persist [18]; moreover, the core microbiota has been associated with host genetics and can be used to predict certain host traits (i.e., methane emissions, rumen and blood metabolites and milk production), making it a potential target for rumen manipulation toward a more efficient ruminant production system [22].

## 3. Defining Ecosystem Disturbance

Disturbances are key components of all ecosystems, affecting any ecological system as a whole and spanning a broad range of spatial and temporal scales. Disturbances are inherently diverse and differ in terms of their origin and temporal pattern, leading to equally diverse ecosystem responses [23]. 

Several definitions have been proposed for the concept of disturbance [24]; Grime [25] defined disturbance as a partial or total destruction of biomass, and Sousa [26] extended this definition by adding that disturbances also create opportunities for the establishment of new individuals. Pickett and White [27] proposed a more general definition, stating that disturbance can be defined as any relatively discrete event that disrupts the structure of an ecosystem, community or population and changes either the resource availability or the physical environment. Therefore, disturbances are causal events that can either alter the immediate environment and exert a potential impact upon a community or directly alter it [28]; depending on the magnitude of the disturbance, organisms may be killed or displaced, consumable resources (e.g., living space and nutrients) may be depleted and habitat structure may be degraded or destroyed [29]. 

**Figure 1:**
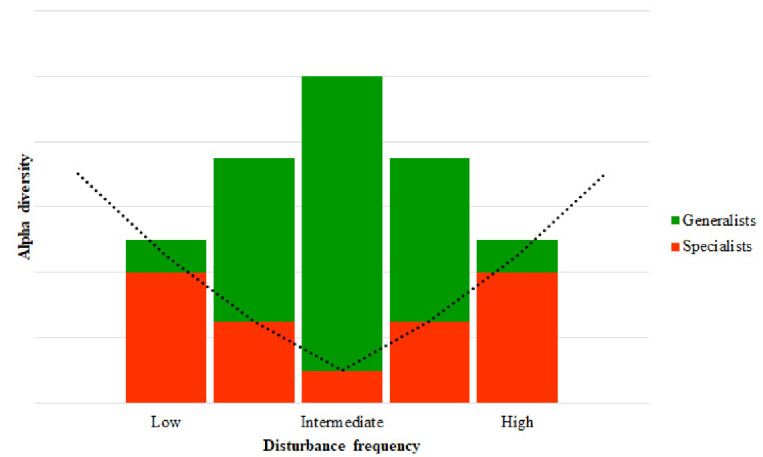
Conceptual representation of the intermediate disturbance hypothesis. The relationship between alpha diversity and disturbance frequency can be understood as follows. At a low frequency of disturbance, strong competitors exclude weaker species, and communities are dominated by few members. An intermediate frequency of disturbance increases rates of mortality, providing an opportunity for inferior species to proliferate. Finally, a high frequency of disturbance may cause excessive rates of mortality, with slow-growth species tending to decrease and disappear. Such a tradeoff between alpha diversity and the rate of disturbance is probably rooted in the processes that drive microbial community assembly. At intermediate rates of disturbance, environmental conditions are unstable, and community assembly is driven by stochastic processes, i.e., all species have equal fitness and chances to succeed; thus, specialized traits are not advantageous for taxa and generalists proliferate, increasing alpha diversity. In contrast, at low and high rates of disturbance, environmental conditions are recurrent, and deterministic processes drive community assembly; more adapted species are selected, so specialists dominate the community, and alpha diversity decreases. Adapted from Svensson et al. [24], Santillan et al. [32] and Sriswasdi et al. [33].

Besides their destabilizing effects, disturbances are believed to play a crucial role in maintaining community robustness; the intermediate disturbance hypothesis [30] predicts maximum diversity at intermediate levels of disturbance frequency (Figure 1).

Disturbances can be classified in four categories, depending on their temporal pattern [28,29,31]. (i) Pulses are short-term and sharply delineated disturbances. (ii) Presses are disturbances that may arise sharply and then reach a constant level that is maintained, evolving into chronic stress. (iii) Ramps are disturbances with steadily increasing or declining strength over time; therefore, they may not have an endpoint or reach an asymptote after an extended period. (iv) Large infrequent disturbances are uncommon events with an extremely low frequency.

This classification can be applied to any disturbance affecting the ruminal microbiota. For instance, in-feed antibiotics administration and frothy bloat episodes are short-term events that are known to perturb the microbial community in rumen (Table 1); thus, they can be categorized as pulses. High-grain diets that lead to subacute ruminal acidosis, as well as the inclusion of certain plant secondary compounds (e.g., tannins, essential oils and saponins) in ruminant diets, can act as a source of chronic stress for the ruminal microbiota (Table 2); thus, such dietary factors are considered presses. In ecology, the incremental spread of an exotic organism is considered a ramp disturbance that the primary community must deal with [29]; analogously in ruminants, the administration of live microorganisms, such as probiotics or rumen content transplants from donor animals, can lead to a progressive increase in incoming microbes, acting as a ramp disturbance for the original microbiota (Table 3). Finally, the complete exchange of rumen content from donor animals for research or other purposes can be regarded as a large infrequent disturbance in the ruminal microbiota (Table 4).

## 4. Defining Microbial Community Robustness

The concept of robustness is widely applied in the scientific literature, although there is considerable confusion about its meaning [53]. In general, robustness is used as a comprehensive term to evaluate the stability of a microbial community, i.e., it describes the extent to which a community exhibits (i) resistance, (ii) resilience or (iii) functional redundancy [54].

Resistance is the ability of a community to remain unchanged in the face of a disturbance and is presumably rooted in the high degree of metabolic flexibility and physiological tolerance of certain microbial populations in response to changing environmental conditions [55]. Evidence suggests that the ruminal microbiota is resistant to various feeding frequencies; in a recent study with cows fed a total mixed ration once or twice a day, the authors did not observe differences in either alpha or beta diversity of the ruminal microbiota between the two treatments [56].

Resilience is the ability of a community to return to its initial structure following a disturbance. Resilience is thought to be a common feature of microbial communities due to some of the intrinsic characteristics of their members. (i) Many microbes have fast growth rates, so if their abundance is suppressed by a disturbance, they have the potential to recover quickly. (ii) Many microbes have a high degree of physiological flexibility, so even if their relative abundance decreases initially, these taxa may acclimate to the new environmental conditions over time and eventually return to their original abundance. Finally, (iii) rapid genetic evolution (through mutations and horizontal gene exchange) can also allow microbial taxa to adapt to new environmental conditions and recover from disturbances [55]. Previous literature reports that the ruminal microbiota is resilient to diverse disturbances. Li et al. [57] tested temporal changes in the rumen microbial community in response to a 72 h butyrate infusion; exogenous butyrate disturbance resulted in significant changes in abundance of four of the five most abundant phyla (Bacteroidetes, Firmicutes, Fibrobacteres and Spirochaetes), but their abundance returned to predisturbance levels 168 h after withdrawal. In another study, Petri et al. [58] evaluated changes in the rumen epimural microbiota induced by diet and an acidotic challenge and observed that all genera undergoing significant shifts in their abundance returned to prechallenge levels during the 1-week recovery period.

Functional redundancy is the ability of a community to maintain or recover its initial function despite compositional changes [54]. Changes in microbial composition may not alter ecosystem process rates for two reasons: (i) the new community might contain taxa that are functionally redundant with respect to the taxa in the old community, and (ii) taxa in the new community may function differently but result in the same process rate when combined at the community level [55]. Determining the functional redundancy inherent to ruminal microbiota is not straightforward; many ruminal species display a broad range of degradative capabilities, making it difficult to discern which particular function of any species might be carried out in situ in the presence of a large number of potential competitors and symbionts [3]. Despite such difficulty, some evidence suggests that the ruminal microbiota is functionally redundant; the division of the number of microbial species by the number of degradable substrates results in many species that can potentially participate in the degradation of each substrate. Moreover, the vast literature on ruminal microbiology and fermentation shows that considerable changes in the microbial community composition often do not translate into changes in fundamental fermentation parameters, such as pH or volatile fatty acid profile [59,60]. 

When a microbial community is not robust enough to cope with a given disturbance, it may evolve to an alternative equilibrium or alternative stable state. The literature has long supported the idea that communities can be found in one of several possible alternative stable states, which is usually referred to as the stability landscape [61]. When alternative stable states occur, state variables that characterize a community (e.g., species composition or function) can persist in one of various possible configurations that constitute different locally stable equilibrium points [62]. The stability landscape concept was first applied in the field of human gut microbiology in 2011, leading to the definition of the term enterotype, which refers to the stratification of the gut microbiota into clusters of microbes of similar nature that can be associated with specific host phenotypes [63]. A few years later, this concept was extended to the ruminal microbiota and referred to as ruminotype by Kittelmann et al. [64]; in their study, the authors characterized three microbial clusters in the rumen differing in their methane yield: ruminotype Q was associated with a reduced ruminal-acetate-to-propionate ratio and had an increased abundance of propionate-producer *Quinella ovalis*; ruminotype S was rich in lactate- and succinate-producing bacteria, such as *Fibrobacter* spp., *Kandleria vitulina*, *Olsenella* spp., *Prevotella bryantii* and *Sharpea azabuensis*; finally, ruminotype H was linked to increased methane emissions and harbored many hydrogen producers, such as *Ruminococcus*, other Ruminococcaceae, Lachnospiraceae, Catabacteriaceae, *Coprococcus*, Clostridiales, *Prevotella*, Bacteroidale and Alphaproteobacteria.

The stability landscape concept is gaining interest with respect to the study of ruminal microbiota, as the existence of alternative stable states or ruminotypes may explain the immense variability observed within and among individual microbial communities [28]. Various factors, such as environment [65], early-life events [66], diet [67], host genetics [68] or even stochastic forces [18], can drive ruminal microbiota assembly and ultimately determine the dominance of an alternative state relative to other states. Community alternative stable states can be differentiated by the enrichment of certain taxonomic or functional groups and do not necessarily imply complete exclusion between them; different taxa and function rates coexist within the rumen microbiota, but the balance among them shifts in each stable state. In that sense, as microbial community states may have diverse functionalities, they could also be connected to many host attributes [69]; for example, in the case of ruminal microbiota, available literature agrees on the existence of three ruminotypes that are associated with differences in animal methane yields [64,70].

## 5. Calculating Microbial Community Robustness

**Figure 2:**
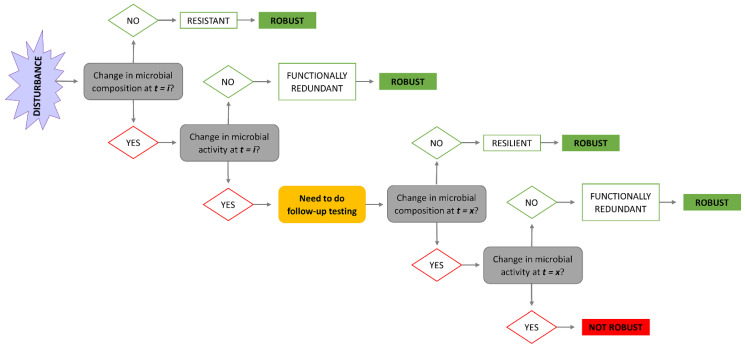
Schematic representation of microbial community robustness determination immediately after disturbance (*t = i*) and at a certain time point after the disturbance (*t = x*).

The estimation of microbial community robustness relies on the measurement of its composition and function stability, i.e., the quantification of its resistance, resilience or functional redundancy, either immediately following a disturbance or at a given time point following a disturbance (Figure 2).

Community resistance, resilience or functional redundancy can be assessed by comparing community variables between samples taken pre- and post-disturbance. Moreover, disturbed communities are often compared to undisturbed control communities to account for any change occurring over time and not due to the disturbance itself. Such calculations are usually presented as a proportion or percentage of the variable measured in the disturbed community vs. the variable measured in the control community at a given time since the disturbance (Table 5). 

The variables that need to be measured to characterize a community and estimate its robustness differ in their nature [71]. Resistance and resilience refer to the composition of the microbial community and can be characterized using any metric of taxon or gene composition, diversity or abundance via a range of molecular or cultural techniques. In contrast, functional redundancy refers to the microbial community activity and can be evaluated through direct assessment of enzyme activities that mediate a process, monitoring of end product accumulation or gene expression analysis (i.e., metatranscriptomics).

## 6. Drivers of Microbial Community Robustness

Previous literature has highlighted that certain attributes of microbial communities, such as alpha diversity and network complexity, may play a crucial role in their ability to respond to disturbances [80,81].

### 6.1. Microbial Alpha Diversity and its Temporal Succession

The first evidence of the positive correlation between ecosystem diversity and stability was obtained in grassland field communities; researchers gathered information about species richness and community biomass over time and found that diversity within an ecosystem tends to exhibit a positive correlation with plant community stability, as it tends to decrease the coefficient of variability in community biomass [82].

The positive effect of diversity on community robustness was later demonstrated in microbial communities. Van Elsas et al. [83] reported a negative correlation between soil microbial richness and survival of invading species, demonstrating that community richness is crucial for preventing the spread of bacterial invaders. Likewise, Wittebolle et al. [84] used microbial microcosms to study denitrifying bacteria and showed that any community should have an even distribution among its members if it is to respond rapidly to disturbances; in contrast, uneven communities excessively depend on their dominant species, and their stability is therefore endangered by environmental fluctuations. 

Community diversity, in terms of both species richness and evenness, enhances ecosystem robustness, probably due to its buffering effect against disturbances, a phenomenon referred to as the insurance hypothesis [81]. The ability of a community to buffer disturbances, loss of species and species invasion is dependent on (i) the functional redundancy of its members and (ii) the ability of its members to respond differentially to disturbances. On the one hand, increasing the community richness increases the odds for functionally redundant and disturbance-resistant species to exist; on the other hand, increasing the community evenness increases the odds that such species can proliferate when disturbances occur [28,84,85]. 

**Figure 3:**
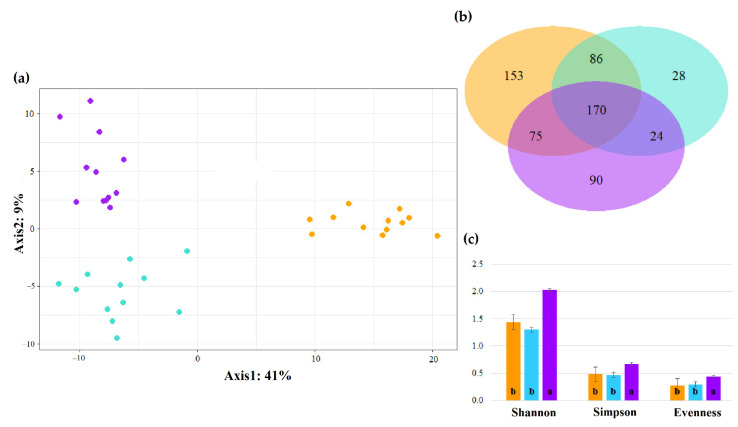
Visualization of the species–time relationship in the ruminal microbiota of 3-month-old (orange), 5-month-old (turquoise) and 9-month-old (purple) fattening bulls (own data). (**a**) Graphical representation of a multivariate analysis (PLS-DA) of bacterial and archaeal taxa in the ruminal fluid. Each point represents a different animal, and the distance between two points indicates the similarity of animal (short distance, similar; long distance, dissimilar). Samples are clearly clustered by age group, highlighting the existence of shifts in microbiota composition relative to time. PERMANOVA test results confirmed that the foreseen graphical differences were significant (*p* < 0.001). (**b**) Venn diagram showing the number of bacterial and archaeal taxa that are shared or unshared by age group, depending on overlaps. Although a core ruminal microbiota exists across age groups, there are unique taxa that change with time. (**c**) Histogram showing the evolution of microbial alpha diversity indices over time, mean values within index with unlike letters differ (*p* < 0.05); the age-dependent increase in ruminal microbiota alpha diversity has already been observed elsewhere [19,20].

When attempting to predict community robustness based on species diversity, it is important to be aware of the possibility that community composition may be sensitive to time, a phenomenon known as the species–time relationship [86,87] (Figure 3). Such patterns of temporal succession of species composition and abundance have been observed not only in animal and plant communities [88,89] but also in microbial communities [90]. The gut microbiota constitutes a clear example of a microbial community in which consistent temporal variability takes place, as previous studies have evidenced in a wide range of hosts, including humans [91], ruminants [19], crustaceans [92] and insects [93].

### 6.2. Microbial Network Complexity

Despite its generalized use, microbial diversity may not necessarily be informative or sensitive enough as an indicator of community state in response to disturbances [94]. When a disturbance occurs, biotic interactions are the first to be affected and can alter the community functioning, even before the species disappear [95]. Some studies have outlined the importance of studying how populations interact with each other to determine the whole community state and functioning [96,97].

It is widely known that individuals establish symbiotic interactions that exist in a continuum from beneficial to antagonistic associations. Cooperation is an association in which two individuals derive benefit from one another; commensalism includes relationships in which one partner derives benefit from the other and the other partner is neither harmed nor benefits from the association; and parasitism is an association whereby one partner (the parasite) lives on or in another partner (the host) and causes harm, as the parasite obtains all or part of their necessary nutrients at the expense of the host. Other antagonistic interactions include competition for a common resource and predation of one individual upon another [98]. All these relationships between individuals in a given community can be represented in networks, providing a single holistic vision of communities that integrates both direct and indirect effects of disturbances on diversity, taxonomic composition and relationships between populations [94]. 

It has long been hypothesized that network complexity may affect community robustness [80]; recently, studies have showed that when an environmental disturbance occurs, microbial network complexity is reduced [99,100]. Karimi et al. [94] and Tylianakis et al. [101] reviewed certain attributes of network structure that may be useful to evaluate community robustness.

1. The number of nodes is the number of connected taxa within the network;
2. The number of edges refers to the number of links established between nodes, and connectance is the number of potential links that are actually realized. An increased number of edges can stabilize the rate of ecosystem processes over time under fluctuating environmental conditions, owing to the ecological redundancy of links, a phenomenon that can also be explained by the previously mentioned insurance hypothesis;
3. Nestedness is the tendency of nodes to interact with subsets of the interaction partners of better-connected nodes; in other words, a network is nested when the species interacting with specialists comprise a proper subset of the species interacting with generalists. Nestedness is an important feature of robust communities in that specialists are usually the first species to go extinct from a network; however, if nested, the remaining species will still have generalists to interact with;
4. The type of interactions between species plays a role in community robustness. Cooperation between species might facilitate colonization but also create dependency and potential mutual downfall, reducing ecological stability. Although competition may drive inefficiencies, it dampens the destabilizing effects of cooperation, increasing overall stability [102];
5. The pattern of interaction strength is also believed to affect community stability; in particular, the presence of many weak links within a network serves to limit energy flow in a potentially strong consumer–resource interaction and, therefore, to inhibit runaway consumption that destabilizes the community dynamics [85];
6. Modularity compartmentalizes networks into subsets in which species interact frequently with one another but minimally with other species outside the compartment. Modularity increases community robustness because disturbances spread more slowly through a modular network; therefore, compartmentalized communities will deteriorate more gradually than randomly connected communities [103];
7. The node degree is the number of interactions established per node, and its distribution is an important parameter determining community robustness; if the interactions are not evenly distributed across nodes within the network and a few well-connected species concentrate most of the existing links, such a community will be robust to random loss of nodes but very fragile to the elimination of the most connected nodes [104].

### 6.3. Potential Modulation of Robustness through Diet

High alpha diversity and increased network complexity are two biological characteristics attributed to robust microbial communities; furthermore, it is known that diet acts as a deterministic effect shaping the ruminal microbiota [18] suggesting that certain dietary strategies could modulate robustness attributes, playing a role in the stability of ruminal microbiota. 

In relation to alpha diversity, it is hypothesized that high-forage diets can increase rumen microbiota alpha diversity because high-concentrate diets promote low pH values, which inhibit the growth of some acid-sensitive rumen bacteria [105]. Such a positive effect of high-forage diets on ruminal microbiota alpha diversity has been observed in multiple ruminant hosts, such as adult [106] and growing [107,108] cattle, sheep [109,110], goats [111] and red deer [112]. A short retention time is expected to reduce microbiota diversity, as it selects for only fast-growing taxa [105]. In general, increasing the feed intake level does reduce rumen retention time [113], with a quadratic effect on the microbial community alpha diversity. Wang et al. [114] observed that microbiota richness and Shannon index reached their highest values when animals were fed 96% of their nutrient requirements, whereas both values decreased when animals were overfed (108% and 120%). Similarly, large and low-density dietary particles have longer retention times in rumen [115,116], although this does not always translate into an increase in microbial community diversity [117]. 

There is no clear picture of how diet can drive microbial network complexity in the rumen. Some studies have shown the influence of dietary protein or energy limitation on microbial co-occurrence networks. Costa-Roura et al. [118] observed that dietary protein reduction increased network complexity in terms of the number of nodes and edges, as well as betweenness centrality. Similarly, Park et al. [119] reported that the number of nodes and edges exclusive to one treatment was increased when dietary energy was limited. With respect to high-forage diets, evidence of reviewed studies agree that including a higher proportion of forage in ruminants’ diet has a positive effect on microbial network complexity in the rumen [107,109]. Supplementation with solid feed during the lactation period can also increase microbial network nestedness in the rumen, as demonstrated in a study of goat kids [120]. Finally, the effects of plant secondary compounds on microbial network complexity depend on the specific compound used. Patra et al. [42] observed a reduction in network complexity in terms of the number of nodes, edges and unique correlations in association with varying doses of menthol-rich plant lipid compounds in growing sheep. In contrast, Popova et al. [44] did not observe any effect on microbial co-occurrence network complexity following administration of tea saponins in dairy cattle. 

In light of the abovementioned results, high-forage diets seem to be the most reliable strategy to increase both alpha diversity and network complexity of the ruminal microbiota. Attention to not overfeeding animals in order to avoid a decrease in rumen retention time, as well as a moderate restriction of dietary energy and protein contents, also appears to be suitable options to enhance microbial diversity and interactions.

## 7. Future Research: The Link between Diet, Ruminal Microbiota Robustness and Host Health

Extensive work in the human gut microbiota has revealed that microbes play essential roles in host metabolism and health [121,122]; similarly, it is widely hypothesized that the microbial community in the rumen influences ruminant health and performance [123,124,125]. However, it is difficult to define a normal or healthy microbiota, as the composition of the rumen microbial community is diverse and highly variable, depending on diet, host and age [19,21]; alternatively, robustness has been proposed as a surrogate marker of a healthy microbial community [126,127]. 

As mentioned above, the main biologic characteristics attributed to robust microbial communities are increased microbial alpha diversity and network complexity. Recent literature has reported that those parameters could be modified by diverse feeding practices (Section 6.3), suggesting that diet could play a crucial role as a modulator of ruminal microbiota robustness. Future research identifying the specific nutritional practices that can enhance or impair community robustness in the rumen would be of considerable interest. 

To date, little evidence has been published to support the link between microbiota robustness and host health. In human studies, reduced microbiota alpha diversity has been associated with a bloom of the opportunistic pathogen *Enterobacter cloacae* after antibiotic administration [128]; similarly, microbiota network complexity has been positively associated with Chron disease remission after ileocolonic resection [129]. Considering the considerable negative impact of digestive disorders on beef cattle production, explaining between 30–42% of the monthly mortality rates in North America [130], further studies accurately determining the real link between ruminal microbiota robustness and animal health would be of central importance. Long-term experimental trials that apply a disturbance to animals with either robust or fragile microbial communities in rumen, following up the posterior shifts in microbiota composition and function, are needed to elucidate the practical benefits of building a more robust rumen. 

**Figure 4:**
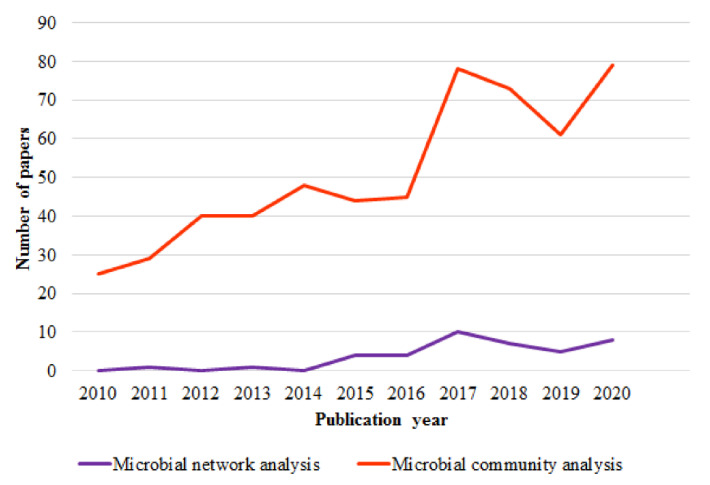
Number of papers reporting microbial community analysis and microbial network analysis in the rumen. Data are based on the Web of Science database (time span: 2010–2020; keywords: “microbial network analysis rumen” and “microbial community analysis rumen”).

To succeed in the evaluation of microbial community robustness in the rumen, it is of vital importance to study both its alpha diversity and network complexity. In that sense, it is worth to highlight that although the number of articles studying the rumen microbial community has increased in the recent years, studies including microbial network analysis are relatively scarce (Figure 4); therefore, there is room for further improvements in the routine analysis of ruminal microbiota sequence data in order to provide a full description of its composition, functioning and robustness. 

## 8. Conclusions

Understanding ruminal microbiota robustness is of considerable importance to predict a community’s response to disturbances. Microbiota robustness depends on its resistance, resilience and functional redundancy, and both increased alpha diversity and network complexity are considered potential drivers of community robustness. Diverse feeding practices have been shown capable of shaping the ruminal microbiota, including its alpha diversity and network complexity; thus, more work is needed to identify nutritional modulators that can enhance or impair community robustness. In addition, more research should be conducted to confirm the real link between microbial community robustness in the rumen and animal health in order to elucidate the practical benefits of building a robust rumen. To these ends, both microbial alpha diversity and network complexity information need to be routinely reported in ruminal microbiota studies, providing a holistic vision of the community.

## Figures and Tables

**Table 1:** Main effects of pulse disturbances on ruminal microbiota (bacteria and archaea).

Disturbance	Animals	Treatment	Effects on Ruminal Microbiota ^1^	Reference
In-feed antibiotic administration	Crossbred steers (finishing)	NA: non-hormone, non-antibiotic treatment AB: hormone-implanted cattle fed a beta-agonist (ractopamine) and antibiotics (monensin, 478.3 g/ton; tylosin, 96.1 g/ton) during the finishing period	Decreased alpha diversity in AB steers (reduced Shannon H and inverse Simpson indices and richness). Beta diversity analysis showed no differences in microbial community composition between NA and AB steers. At the phylum level, no differences in taxon abundance between NA and AB steers. At the genus level, decreased abundance of Bacteroidetes (*Spirosoma, Dyadobacter, Leadbetterella* and *Zunongwangia*) in AB steers. Gram-positive Firmicutes were partially replaced by Gram-negative Negativicutes in AB steers.	[34]
In-feed antibiotic administration	Holstein cows (pluriparous)	CTR: non-antibiotic treatment MO: administration of 335 mg monensin/d during the transition period	Decreased alpha diversity in MO cows (reduced values of Shannon index and richness). Beta diversity analysis showed significant differences in microbial community composition between MO and CTR cows. Decreased abundance of 23 bacterial OTUs in MO (mainly belonging to Bacteroidetes and Firmicutes phyla). Increased abundance of 10 bacterial OTUs in MO (belonging to Actinobacteria, Bacteroidetes, Cyanobacteria and Firmicutes phyla). No difference between CTR and MO cows in archaeal abundance (i.e., *Methanobrevibacter*).	[35]
Frothy bloat	Steers	Steers grazed on winter wheat and were visually scored for bloat: BS0: normal; no visible signs of bloat BS2: marked distention of left side of animal; rumen distended toward top of back	Increased abundance of archaeal community and decreased abundance of bacterial community in BS2 steers. At the bacterial phylum level, increased abundance of Firmicutes and Proteobacteria but decreased abundance of Bacteroidetes and Actinobacteria in BS2 steers. Among archaea, increased abundance of *Methanobrevibacter* but decreased abundance of *Methanosphaera*, *Methanosarcina*, *Methanocorpusculum*, *Methanococcus* and *Methanococcoides* in BS2 steers. Decreased number of interactions among both bacteria and archaea in BS2 steers.	[36]
Frothy bloat	Angus steers (3–4 years)	PA: pure alfalfa pasture AA: pure alfalfa pasture, but steers treated with detergent AS: mixed alfalfa–sainfoin pasture Steers were visually scored for bloat: NB: non-bloated steers B: slightly to severely bloated steers	Increased alpha diversity in rumen solid fraction of B steers (increased values of Shannon index and richness). No effect of bloat on alpha diversity in rumen liquid fraction. Beta diversity analysis showed that rumen solid fraction microbiota composition differed between B and NB steers. Beta diversity analysis showed that rumen liquid fraction microbiota composition differed between PA-B and AA-NB and tended to differ between PA-B and AS-NB steers. At the genus level, increased abundance of *Succinivibrio* and *Streptococcus* but decreased abundance of *Fibrobacteres* and *Ruminococcus* in B steers.	[37]

^1^ Alpha diversity measures the variability of taxa within a particular community; it is usually assessed through species richness (the number of species present in a community), Shannon index (based on the weighted geometric mean of the proportional abundances of species, measuring the entropy of a system) and Simpson index (the probability of any two individuals drawn at random from an infinitely large community belonging to different species). Beta diversity measures the variability of taxa between communities, commonly examined via distance/dissimilarity matrices.

**Table 2:** Main effects of press disturbances on ruminal microbiota (bacteria and archaea).

Disturbance	Animals	Treatments ^1^	Effects on Ruminal Microbiota ^1,2^	Reference
Subacute ruminal acidosis	Dairy cows (760 kg)	Sampling protocol lasted for 7 weeks: Week 0: baseline. Week 1 and 3: 65% grain diet. Week 4 and 6: chopped hay diet.	Bacterial density in rumen solids increased during weeks 4 and 6 compared to weeks 1 and 3. Alpha diversity decreased in week 3 compared to week 0 (lower values of Shannon index). Beta diversity analysis showed that ruminal microbiota composition in week 3 was different from week 0 and week 6, but week 0 and week 6 did not differ.	[38]
Subacute ruminal acidosis	Holstein cows (pluriparous, 460 kg)	Crossover design 2 treatments × 2 periods (21 d): COD: 40% concentrate diet. SAID: 70% concentrate diet.	Decreased alpha diversity in SAID cows (lower values of Shannon index and richness). At phylum level, increased abundance of Firmicutes and Actinobacteria whereas decreased abundance of Bacteroidetes, Lentisphaerae and Proteobacteria in SAID cows. At genus level, increased abundance of *Ruminococcus*, *Atopobium* and *Bifidobacterium* whereas decreased abundance of *Prevotella*, *Treponema*, *Papillibacter*, *Anaeroplasma* and *Acinetobacter* in SAID cows. More abundant gram-positive bacteria than gram-negative bacteria in SAID cows.	[39]
Tannins	Holstein cows (584 kg)	CTR: no supplementation. TA: 2 g chestnut and quebracho tannins blend/kg DM for 12 d.	Slight effects on alpha diversity (richness tended to decrease in TA cows). Beta diversity analysis showed no differences between TA and CTR microbial composition in rumen. At phylum level, increased abundance of Firmicutes in TA cows. At genus level, increased abundance of *Ruminococcus*, *L7A-E11*, *Blautia*, *Anaerofustis*, *Anaerovibrio* whereas decreased abundance of *RFN20*, *Fibrobacter*, *Treponema* and *Methanosphaera* in TA cows.	[40]
Tannins	Simmental steers (350 kg)	CTR: no supplementation. TA: 16.9 g tannic acid/kg DM for 5 d.	Increased alpha diversity in TA steers (higher values of Shannon index). Beta diversity analysis showed no differences between TA and CTR microbial composition in rumen. At phylum level, increased abundance of Tenericutes in TA steers. At genus level, increased abundance of *Saccharofermentans* in TA steers.	[41]
Essential oils	Holstein cows (pluriparous)	CTR: no supplementation. EO: 1 g essential oils blend/d containing thymol, guaiacol, eugenol, vanillin, salicylaldehyde and limonene during the transition period.	No effects on alpha diversity. Beta diversity analysis showed significant differences between EO and CTR microbial composition in rumen. No difference between CTR and EO cows in archaeal or bacterial abundance at any taxonomic level.	[35]
Essential oils	Suffolk lambs (121 d, 33 kg)	CTR: no supplementation PBLC-L: 80 mg menthol-rich PBLC/d for 4 weeks PBLC-H: 160 mg menthol-rich PBLC/d for 4 weeks	No effects on alpha diversity. In the rumen solid fraction, increased abundance of *Dehalobacteriaceae*, *Mycoplasmataceae*, UG *Lachnospiraceae,* US *Dehalobacterium,* US *Desulfovibrio* but decreased abundance of *Christensenellaceae*, UG *Paraprevotellaceae*, Euryarchaeota, US *Methanosphaera,* US *Prevotella, LD1-PB3* and UG *LD1-PB3* in PBLC lambs. In the rumen liquid fraction, increased abundance of *WCHB1-25,* US *WCHB1-25*, *Bacteroidaceae*, US *BF311* and US *YRC22* but decreased abundance of Christensenellaceae, UG *Christensenellaceae*, Thermoplasmata, *Methanomassiliiococcaceae*, *vadinCA11*, US *Blautia 2* and UC Proteobacteria in PBLC lambs. Reduced microbial network complexity (fewer edges, nodes and unique interactions) in PBLC lambs.	[42]
Saponins	Holstein bulls (150 d, 150 kg)	AH: concentrate plus alfalfa hay AHS: AH plus 9 g camellia seed saponins/d for 4 weeks SH: concentrate plus soybean hulls SHS: SH plus 9 g camellia seed saponins/d for 4 weeks	No effects on alpha diversity in AH and AHS. Increased alpha diversity (higher values of richness) in SHS bulls compared to SH. Beta diversity analysis showed differences in microbial composition between the four treatments. No difference between AH and AHS bulls in microbial abundance at any taxonomic level. Increased abundance of *Prevotella 1*, *Christensenellaceae R-7*, *Prevotellaceae Ga6A1*, *Clostridium sensu stricto 1*, *Ruminococcaceae UCG-002* and *Prevotellaceae YAB2003 group* bur decreased abundance of *Ruminococcaceae NK4A214* and *Syntrophococcus* in SHS bulls compared to SH.	[43]
Saponins	Holstein cows (658 kg)	CTR: no supplementation TEA: 0.77% tea saponin for 5 weeks	No effects on alpha diversity. Beta diversity analysis showed no differences in microbial composition between CTR and TEA cows. Decreased abundance of UC Deltaproteobacteria in TEA cows. Slight reduction in microbial network complexity (fewest edges) in TEA cows.	[44]

^1^ DM, dry matter; PBLC, plant bioactive lipid compounds; UC, unclassified; UG, unclassified genus; US, unclassified species; ^2^ Please see footnote in Table 1.

**Table 3:** Main effects of ramp disturbances on ruminal microbiota (bacteria and archaea).

Disturbance	Animals	Treatments	Effects on Ruminal Microbiota ^1,2^	Reference
Probiotic administration	Crossbred steers (434 kg)	CTR: no probiotic administration. P169: administration of *Propionibacterium acidipropionici* strain P169 (10^11^ cfu/d) during the finishing period.	No effects on alpha diversity. Beta diversity analysis showed no differences in microbial community composition between CTR and P169 steers. Increased gene copy numbers of *Propionibacterium acidipropionici* strain P169 in P169 steers by qPCR. At phylum level, no differences in taxon abundance between CTR and P169 steers. At genus level, increased abundance of *Phascolarctobacterium*, UC Clostridiaceae and Lachnospiraceae, whereas decreased abundance of *Prevotella*, *Succinivibrio*, *YRC22* and UC Veillonellaceae in P169 steers.	[45]
Probiotic administration	Romane lambs (fattening)	CTR: no probiotic administration. SUP: administration of a combination of live yeast *Saccharomyces cerevisiae* CNCM I-1077 and selected yeast metabolites in milk replacer (3 × 10^9^ cfu/d plus 0.45 g yeast metabolites/d) and in feed (6 × 10^6^ cfu/g plus 1.5 kg yeast metabolites/ton) during the whole fattening period.	No effects on alpha diversity. Beta diversity analysis showed no differences in microbial community composition between CTR and SUP lambs. At OTU level, increased abundance of *Snodrgrassella, Megasphaera, Bifidobacterium, Butyricimonas, Succinivibrio* and *Fibrobacter*, whereas decreased abundance of *Desulfovibrio* and *Bacteroides*.	[46]
Probiotic administration	Holstein steers (504 kg)	CON: no probiotic administration. YEA: administration of a feed additive containing *Saccharomyces cerevisiae* and other active ingredients from yeast cell wall (15 g commercial product/d for 25 d).	No effects on alpha diversity. Beta diversity analysis showed significant differences in microbial community composition between CON and YEA steers. At phylum level, increased abundance of Saccharibacteria in YEA steers. At genus level, increased abundance of *Ruminococcaceae NK4A214, Christensenellaceae R-7, Ruminococcaceae UCG-010, Candidatus Saccharimonas, Bacteroidales BS11 gut group, Ruminococcus 2, Anaerovorax, Lachnospiraceae UCG-008* and *Ruminococcaceae UCG-005*, whereas decreased abundance of *Lachnoclostridium, Lachnoclostridium 5* and *Bacillus* in YEA steers.	[47]
Probiotic administration	Jintang black male goats (80 d)	CTR: no probiotic administration BA: administration of *Bacillus amyloliquefaciens* fszne-06 (10^9^ cfu every 2 d for 30 d) BP: administration of *Bacillus pumilus* fszne-09 (10^9^ cfu every 2 d for 30 d)	Increased alpha diversity (increased values of Shannon and Simpson indices and richness) in BA and BP goats. Beta diversity analysis showed significant differences in microbial community composition between treatments. At the phylum level, increased abundance of Firmicutes in BA and BP goats and decreased abundance of Bacteroidetes in BA goats. At the genus level, increased abundance of *Succiniclasticum* in BA and of UC *Ruminococcaceae* in BP goats. Decreased abundance of *Klebsiella* in BA and BP goats.	[48]
Inoculation with rumen content from donor animals	Donors: Hu sheep (36 kg) Recipients: Hu lambs (1–28 d)	C: non-transfaunated lambs. IBW: lambs transfaunated before weaning (20 mL of sheep ruminal fluid mixture via stomach tube, four inoculations) IDW: lambs transfaunated during weaning (20 mL of sheep ruminal fluid mixture, two inoculations)	Increased alpha diversity in donor ruminal fluid mixture did not translate to increased alpha diversity in transfaunated lambs. Beta diversity analysis showed no differences in microbial community composition between treatments. At the genus level, increased abundance of *Prevotellaceae UCG-001, Moryella, Succiniclasticum* and *Tyzzerella 4* in IBW lambs compared to C and increased abundance of *Erysipelatoclostridium, Eubacterium coprostanoligenes* and *Sharpea* in IDW lambs compared to C.	[49]
Inoculation with rumen content from donor animals	Donors: crossbred cows (adults) Recipients: sheep (1–4 years, 35 kg)	CON: non-transfaunated sheep TRANS: transfaunated sheep (administration of 1.5 L of cow ruminal fluid mixture via stomach tube once)	Increased alpha diversity in donor ruminal fluid mixture did not translate to increased alpha diversity in TRANS sheep. Beta diversity analysis showed changes in community structure but not membership in TRANS sheep, suggesting that the new species introduced by transfaunation were not able to colonize recipient rumen but favored or inhibited growth of some established species. At the phylum level, no differences in taxon abundance between CON and TRANS sheep. At the genus level, decreased abundance of *Selenomonas* in TRANS sheep.	[50]

^1^ UC, unclassified; ^2^ Please see footnote in Table 1.

**Table 4:** Main effects of large, infrequent disturbances on ruminal microbiota (bacteria and archaea).

Disturbance	Animals	Treatments ^1^	Effects on Ruminal Microbiota ^1,2^	Reference
Complete exchange of rumen content from donor animals	Holstein cows (multiparous)	HE: high-efficiency cows exchanged rumen content with low-efficiency cows LE: low-efficiency cows exchanged rumen content with high-efficiency cows Sampling period included: Pre: from 8 to 0 d before exchange. Post 1: from 0 to 10 d after exchange. Post 2: from 10 to 56 d after exchange.	In Pre, LE cows exhibited higher alpha diversity than HE cows. After exchange, HE cows showed increased Shannon index and richness values in Post 1 and returned to pre-exchange levels in Post 2. LE cows had decreased Shannon index values in Post 1 and returned to pre-exchange levels in Post 2, whereas no change in richness values was observed over time. Beta diversity analysis showed significant differences in microbial community composition between treatments. The general trend was that of a donor-like community in Post 1 induced by the exchange and, in contrast to Pre, returned to a community similar to Pre in the host in Post 2.	[51]
Complete exchange of rumen content from donor animals	Holstein cows (multiparous, 582 kg)	SARA induction period: CON: 40% concentrate diet HG: 60% concentrate diet After the SARA induction period, the rumen content transplant period began, and cows in the HG group were categorized as: DR: cows receiving 70% rumen content from CON cows SR: cows receiving 70% self-derived rumen content Sampling period lasted until 20 d after exchange.	Increased alpha diversity (increased values of Shannon index) in DR cows. Beta diversity analysis showed significant differences in microbial community composition between DR and SR, despite samples starting to cluster together 4 d following exchange. At the phylum level, increased abundance of Firmicutes and decreased abundance of Bacteroidetes and Spirochaetes in DR cows. At the genus level, increased abundance of UC *Ruminococcaceae* and Saccharofermentans but decreased abundance of UC *Prevotellaceae* and *Treponema* in DR cows. Microbial network analysis showed that rumen content exchange only affects non-keystone OTUs (i.e., taxa that display weak interactions with other taxa).	[52]

^1^ SARA, subacute ruminal acidosis; UC, unclassified; ^2^ Please see footnote in Table 1.

**Table 5:** Summary of indices to assess changes in community variables following a disturbance.

Changes Immediately Following a Disturbance ^1^	Changes at a Certain Time Point After a Disturbance ^1^	Reference
$\frac{D_{i}}{C_{i}}$	$\frac{D_{x}}{C_{x}}$	[72]
$\frac{C_{i}-D_{i}}{C_{i}}*100$	$\frac{C_{x}-D_{x}}{C_{x}}*100$	[73]
$1-\frac{2*\left|C_{i}-D_{i}\right|}{\left(C_{i}+\left|C_{i}-D_{i}\right|\right)}$	$\frac{2*\left|C_{i}-D_{i}\right|}{\left(|C_{i}-D_{i}|+\left|C_{x}-D_{x}\right|\right)}-1$	[74]
$-100*\left(\frac{C_{i}-D_{i}}{C_{i}}\right)$	$-100*\left(\frac{C_{x}-D_{x}}{C_{x}}\right)$	[75]
$D_{i}-D_{0}$	$\frac{D_{x}}{D_{0}}$	[76]
$\frac{D_{i}}{D_{0}}$	$\frac{D_{x}}{D_{0}}$	[77]
$\frac{D_{0}}{\left|D_{i}-D_{0}\right|}$	$\frac{\left|D_{i}-D_{0}\right|}{\left|D_{x}-D_{0}\right|}$	[78]
$\frac{D_{i}}{D_{0}}$	$\frac{D_{x}}{D_{0}}-\frac{D_{i}}{D_{0}}$	[79]

^1^*C* and *D* are the community variables of an undisturbed control system and a disturbed system, respectively, at a given point in time: *t = 0*, pre-disturbance period; *t =* *i*, a time point immediately following a disturbance; and *t = x*, a certain time point after a disturbance.

## Data Availability

The study did not report any data.
